# Modeling comparative cost-effectiveness of SARS-CoV-2 vaccine dose fractionation in India

**DOI:** 10.1038/s41591-022-01736-z

**Published:** 2022-02-24

**Authors:** Zhanwei Du, Lin Wang, Abhishek Pandey, Wey Wen Lim, Matteo Chinazzi, Ana Pastore y. Piontti, Eric H. Y. Lau, Peng Wu, Anup Malani, Sarah Cobey, Benjamin J. Cowling

**Affiliations:** 1 WHO Collaborating Centre for Infectious Disease Epidemiology and Control, School of Public Health, Li Ka Shing Faculty of Medicine, The University of Hong Kong, Hong Kong Special Administrative Region, Hong Kong, China; 2 Laboratory of Data Discovery for Health Limited (D24H), Hong Kong Science and Technology Park, Hong Kong Special Administrative Region, Hong Kong, China; 3 Department of Genetics, University of Cambridge, Cambridge, UK; 4 Center for Infectious Disease Modeling and Analysis, Yale School of Public Health, New Haven, CT USA; 5 Laboratory for the Modeling of Biological and Socio-technical Systems, Northeastern University, Boston, MA USA; 6 Law School, University of Chicago, Chicago, IL USA; 7 Department of Ecology and Evolutionary Biology, University of Chicago, Chicago, IL USA

**Keywords:** Epidemiology, Infectious diseases

## Abstract

Given global Coronavirus Disease 2019 (COVID-19) vaccine shortages and inequity of vaccine distributions, fractionation of vaccine doses might be an effective strategy for reducing public health and economic burden, notwithstanding the emergence of new variants of concern. In this study, we developed a multi-scale model incorporating population-level transmission and individual-level vaccination to estimate the costs of hospitalization and vaccination and the economic benefits of reducing COVID-19 deaths due to dose-fractionation strategies in India. We used large-scale survey data of the willingness to pay together with data of vaccine and hospital admission costs to build the model. We found that fractional doses of vaccines could be an economically viable vaccination strategy compared to alternatives of either full-dose vaccination or no vaccination. Dose-sparing strategies could save a large number of lives, even with the emergence of new variants with higher transmissibility.

## Abstract (web summary)

A multi-scale model using population-level transmission and individual-level vaccination data suggests that vaccine dose fractionation could be a cost-effective strategy for mitigating the COVID-19 pandemic in India.

## Main

COVID-19 remains a major public health crisis that can overwhelm fragile healthcare and socioeconomic systems, especially in developing countries. As of 14 January 2022, India had reported 36.9 million confirmed cases of COVID-19 and 486,000 deaths. Globally, there were more than 324 million reported cases and 5.53 million deaths^1^.

To mitigate the COVID-19 pandemic, various non-pharmaceutical interventions (for example, travel restrictions, quarantine of cases and contacts, social distancing and lockdown) have been repeatedly implemented. However, these measures cannot be maintained for the long term because of the detrimental effect on socioeconomic sustainability and population mental health^2^. It is encouraging that, by 14 January 2022, 114 vaccine candidates had been tested in humans and 47 had entered final phases of clinical trials^3^. Although 4.4 billion people had received at least one dose of COVID-19 vaccine as of the end of 2021, global vaccine shortages and inequity of COVID-19 vaccine distribution remain problematic with no well-established global procurement mechanism^4^. Despite having approximately 82% of the global population, only 11% and 47% of the population in low- and middle-income countries (LMICs^5^) had received one dose or were fully vaccinated, respectively, by 14 January 2022 (ref. ^6^).

India is among the countries worst hit by the COVID-19 pandemic, and it faced a shortage of vaccines in the early outbreak^7^. India began its COVID-19 vaccination program on 16 January 2021 and, by 14 January 2022, had administered 837 million doses, with 46% of the population partially vaccinated and 18% fully vaccinated^8^. As of 14 January 2022, nine COVID-19 vaccines, including ChAdOx1 (Covishield, AstraZeneca), BECOV2A (Corbevax, Biological E. Limited), BBV152 (Covaxin, Bharat Biotech), Gam-COVID-Vac (Sputnik V, Gamaleya) and mRNA-1273 (Spikevax, Moderna), have received emergency use authorization in India^3,9^.

Given global vaccine shortages, dose fractionation of vaccines has been proposed as a potential strategy to accelerate the accrual of population immunity through vaccination^10^. Assuming that vaccines approved in India face a shortfall in the total number of full doses available, we evaluated the economics of potential costs and benefits of dose fractionation of COVID-19 vaccines. Studies of COVID-19 mRNA vaccines indicate that fractional doses could still provide a robust immune response against COVID-19 (ref. ^11^). For the mRNA-1273 vaccine, two doses of 25 µg elicited about half the geometric mean PRNT_80_ titers (which is a measure widely used to evaluate the neutralizing antibody responses against viruses) at 14 days compared to two doses of the standard dose (100 μg)^11^. Recent modeling studies suggest that dose-sparing strategies can reduce the disease burden of COVID-19 as compared to the full-dose strategy^12,13^. Moreover, fractional-dose strategies have been used successfully in the past to address vaccine shortages for inactivated poliovirus vaccines^14^ and meningococcal conjugate vaccines in outbreak events^15^. Based on lengthy experience with 17DD yellow fever, knowledge of the effect of fractional doses and durable responses with this vaccine, one-fifth of the standard dose of the 17DD yellow fever vaccine was used in Angola and the Democratic Republic of Congo to accelerate vaccine rollout during their 2016 outbreaks^16,17^.

**Fig. 1:**
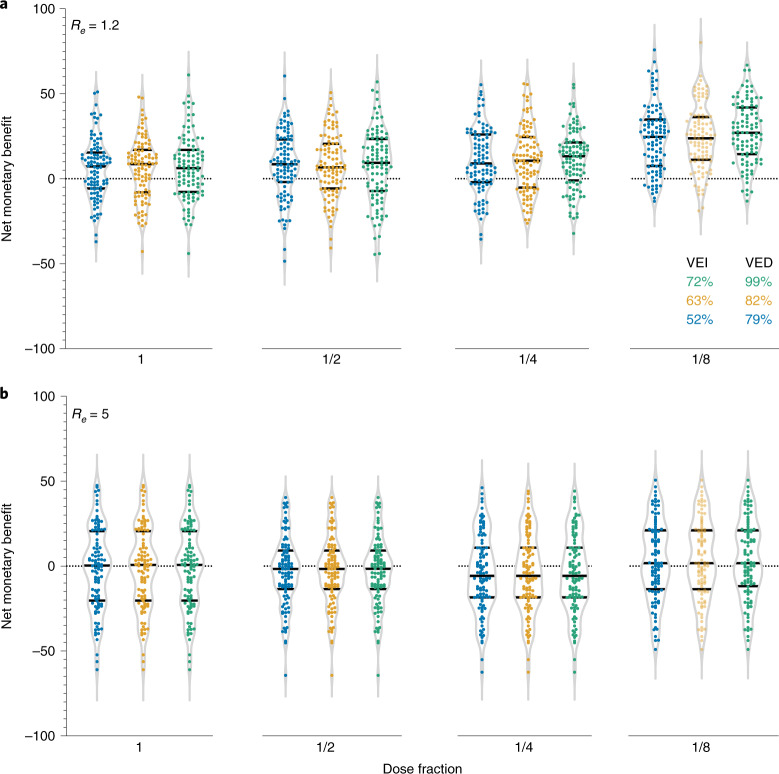
Estimation of the expected gain in the NMB for each vaccination strategy as compared to the status quo strategy (that is, no vaccination) in India. **a**, Estimation given the effective reproductive number *R*_*e*_ = 1.2. **b**, Estimation given *R*_*e*_ = 5. In **a** and **b**, each sub-panel from left to right corresponds to the two-dose vaccination using full dosing, 1/2 dosing, 1/4 dosing and 1/8 dosing, respectively. Each dot indicates one realization of the 100 stochastic simulations. Color scheme indicates the vaccine efficacy against infection (VEI) and vaccine efficacy against symptomatic disease (VED). The WTP per averted YLL per age group is based on data from the Centre for Monitoring Indian Economy’s Consumer Pyramids Household Survey^35^ (Methods). Extended Data Fig. 2 provides the sensitivity analyses using other possible WTP per averted YLL.

**Extended Data Fig. 1:**
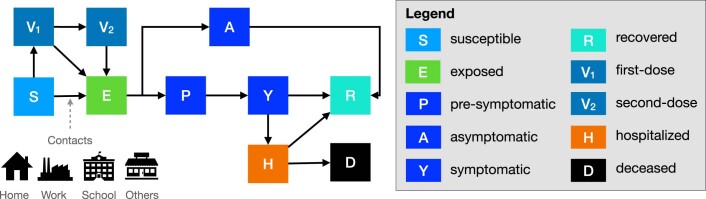
Schematic of the individual-based model of SARS-CoV-2 transmission and vaccination. Following infection from age-specific contacts in home, work, school or others, susceptible individuals (S) become exposed (E), during which they are infected but not yet infectious or symptomatic. After the incubation period, each infected case becomes asymptomatic (A), in which the asymptomatic case has a reduced infectiousness before recovery (R). The remaining infected cases progress to be pre-symptomatic (P), during which they have a moderate infectiousness with no symptoms. The pre-symptomatic cases progress to symptomatic infectious (Y), with a subset becoming hospitalized (H) or deceased (D). Recovered individuals remain permanently protected from future infection. Vaccinated individuals progress to a one dose (V1) followed by a two dose compartment (V2) with different efficacies.

**Extended Data Fig. 2:**
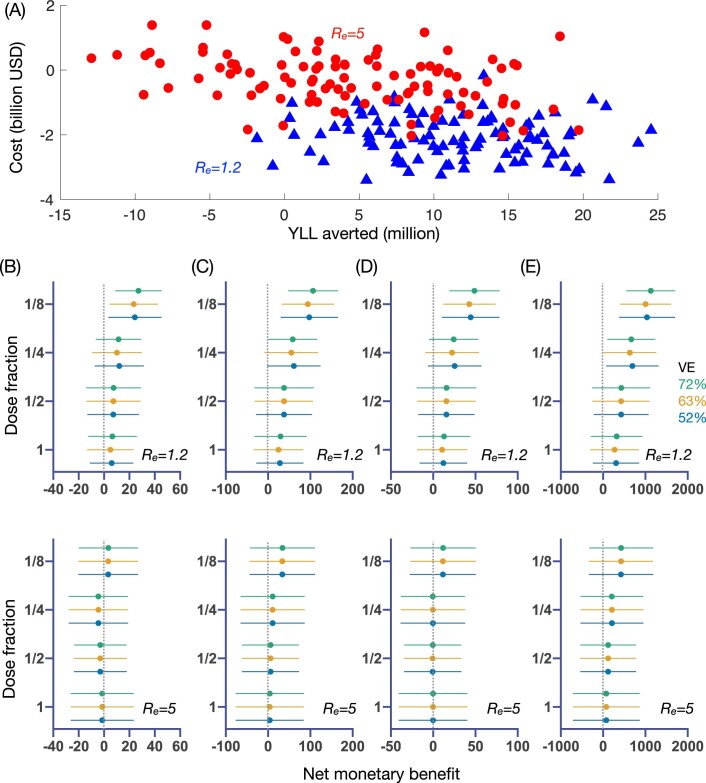
Estimation of the expected gain in the net monetary benefit (NMB) for each fractional-dose strategy as compared to status quo in India. (A) An example for the estimated costs of fractionated dose strategies versus the averted years of life lost (YLLs) with reference to the no vaccine strategy. Each dot indicates one realization of the 100 stochastic simulations, given the use of 1/8 dose fractionation strategy with 72% vaccine efficacy against infection (VE) (blue triangle and red circle indicate the estimates given effective reproductive number *R*_*e*_ = 1.2 and 5, respectively) and willingness to pay (WTP) per YLL averted of US$1,097, US$1,251, US$2,977, US$3,150, and US$3,205 for five age groups (0-5, 6-17, 18-49, 50-64, and > 65), respectively. (B) Estimated distributions for the expected gain in the NMB (2021 $ billion) over various vaccine efficacy against infection and fractional-dose strategy, given vaccine cost of US$12 and willingness to pay (WTP) per YLL averted of US$1,097, US$1,251, US$2,977, US$3,150, and US$3,205 for five age groups (0-5, 6-17, 18-49, 50-64, and > 65), respectively. Dots and error bars indicate the estimated mean and standard deviations, respectively. Colour scheme corresponds to the vaccine efficacy against infection. (C) Same as (B) but setting the WTP per averted YLL as US$10,517. (D) Same as (B) but setting the WTP per averted YLL as US$5089 (i.e., as in other middle-income countries such as Thailand). (E) Same as (B) but setting the WTP per averted YLL as US$100,000 (i.e., as in high-income countries such as theUS). Each subpanel in (B) to (E) uses 100 stochastic realizations to simulate theCOVID-19 transmission over each possible vaccine efficacy against infection (between 52% and 72%) and transmissibility (with effective reproduction number Re = 1.2 or 5). The optimal strategy given each transmission scenario is always estimated to be strategies with higher-fold fractionations, even if the vaccine efficacy against infection is not high. See Methods for the calculation of the NMB and supplementary materials for detailed values of vaccine costs and efficacy parameters.

**Extended Data Fig. 3:**
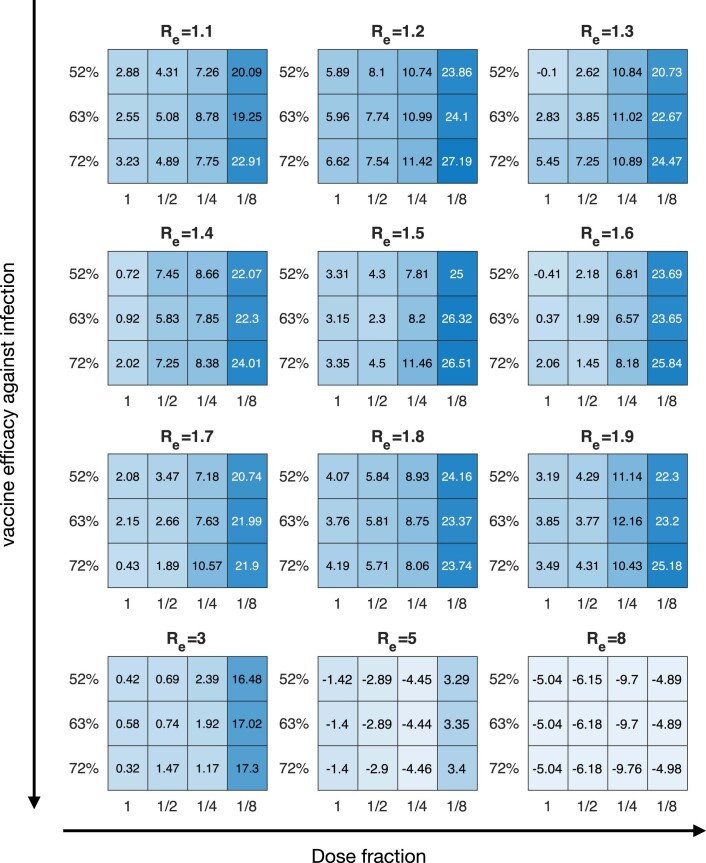
Mean estimation (in 2021 US dollars) of the expected gain in the net monetary benefit (NMB) for each fractional-dose strategy as compared to the status quo strategy (i.e., no vaccination) in India. The estimation examines a wide range of possible transmission (with *Re* increasing from 1.1 to 8) and vaccine efficacy (Supplementary Table 2) scenarios. Given each *Re*, the vaccine efficacy (VE) against infection and VE against symptomatic disease are considered as 52% and 79% in the top row, 63% and 82% in the middling row, and 72% and 99% in the bottom row in each subpanel. The vaccine cost in each strategy is considered as in Supplementary Table 4, and the WTP per YLL averted are US$1,097, US$1,251, US$2,977, US$3,150, and US$3,205 for five age groups 0-5, 6-17, 18-49, 50-64, and > 65, respectively. Under various transmission scenarios, the optimal strategy would always be the vaccination with higher-fold fractionations, even if the VE against infection and against symptomatic disease are not high.

**Extended Data Fig. 4:**
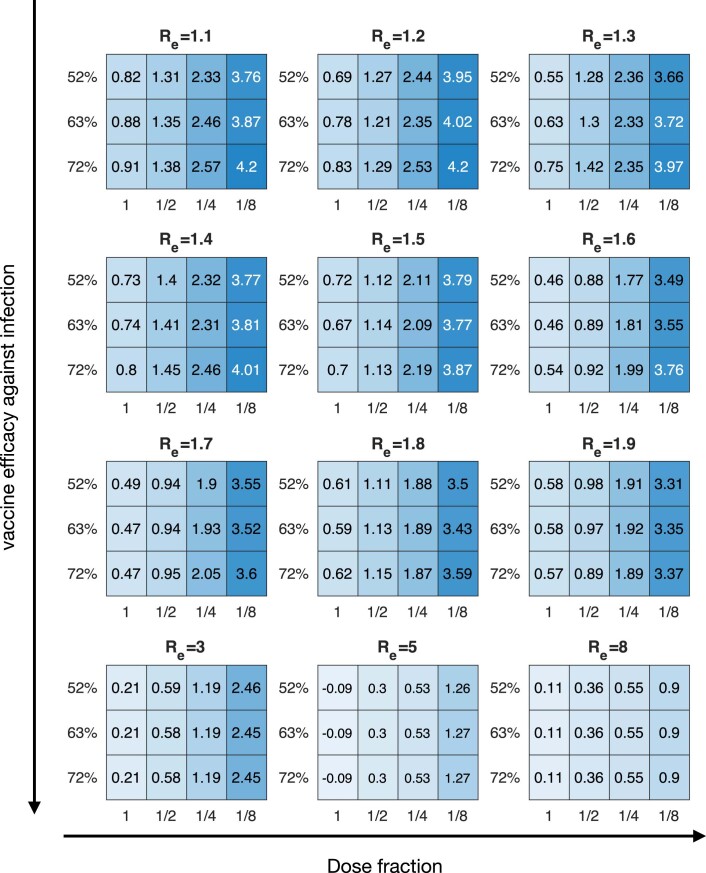
Projected number of hospitalizations averted (million) for each fractional-dose strategy as compared to the status quo strategy (i.e., no vaccination) in India. The estimation examines a wide range of possible transmission (with *Re* increasing from 1.1 to 8) and vaccine efficacy (Supplementary Table 2) scenarios. Given each *Re*, the vaccine efficacy (VE) against infection and VE against symptomatic disease are considered as 52% and 79% in the top row, 63% and 82% in the middling row, and 72% and 99% in the bottom row in each subpanel.

**Extended Data Fig. 5:**
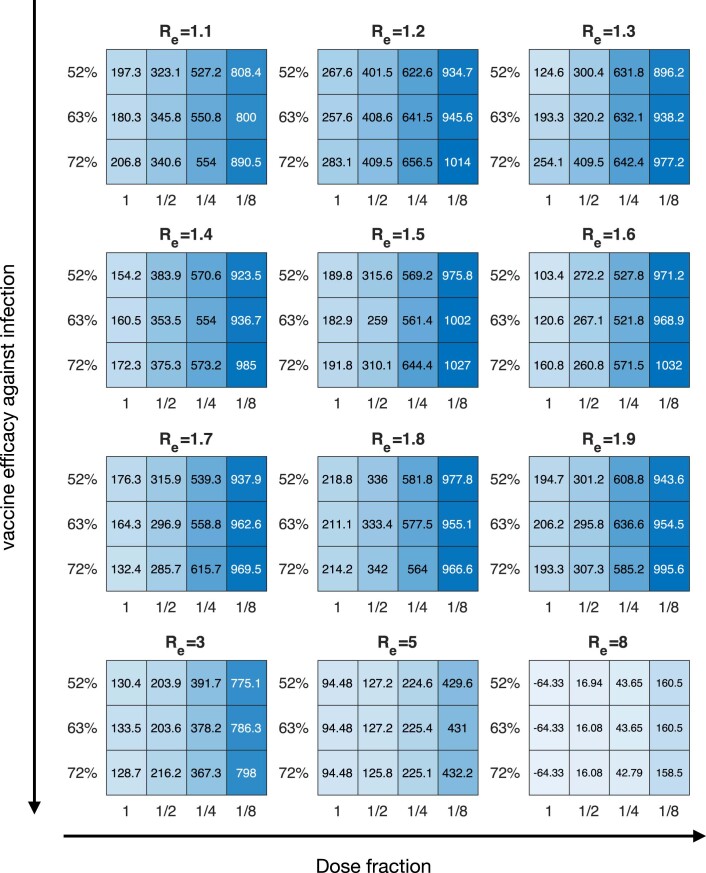
Projected number of deaths averted (thousand) for each fractional-dose strategy as compared to the status quo strategy (i.e., no vaccination) in India. The estimation examines a wide range of possible transmission (with *Re* increasing from 1.1 to 8) and vaccine efficacy (Supplementary Table 2) scenarios. Given each *Re*, the vaccine efficacy (VE) against infection and VE against symptomatic disease are considered as 52% and 79% in the top row, 63% and 82% in the middling row, and 72% and 99% in the bottom row in each subpanel.

The effect of global vaccine shortages is expected to be greatest in LMICs^4^. This situation is worsened by the emergence of new severe acute respiratory syndrome coronavirus 2 (SARS-CoV-2) variants (for example, Alpha (B.1.1.7), Beta (B.1.351), Gamma (P.1), Delta (B.1.617.2) and Omicron (B.1.1.529)), against which vaccine effectiveness might be reduced^18^. Fractionation of vaccine doses could be an effective strategy for mitigating risks while the virus continues to evolve and spread, provided that vaccination provides some protection against new variants^19^. Here we use an individual-based model incorporating household-specific and age-stratified SARS-COV-2 transmission rates coupled with vaccination rollout rates to assess the costs and benefits of fractional-dose vaccines (Extended Data Fig. 1). We considered the costs associated with hospitalization and vaccination and the economic benefits of averting COVID-19 deaths associated with dose-fractionation strategies. We used real-world data to parameterize the vaccine costs and willingness to pay (WTP) per age group (Methods). We explored a wide range of possible disease transmissibility and vaccine efficacy (Fig. 1, Extended Data Fig. 2 and Supplementary Tables 1 and 2). By comparing several potential fractional-dose strategies with alternatives of either full-dose vaccination or no vaccination, we found that vaccination with higher-fold fractionations will increase the cost-effectiveness, even if vaccine efficacy against infection is not high (Fig. 1 and Extended Data Figs. 2–5). The main findings and limitations of the study are summarized in Table 1.

**Table 1:** Policy summary

Background	Given global vaccine shortages and inequity of vaccine distributions, fractionation of vaccine doses might be an effective strategy for reducing public health and economic burden, notwithstanding the emergence of COVID-19 variants. During previous outbreaks, fractional-dose strategies were applied to alleviate vaccine shortages for inactivated poliovirus vaccines, meningococcal conjugate vaccines and yellow fever vaccines.
Main findings and limitations	We developed a multi-scale model incorporating population-level transmission and individual-level vaccination to evaluate the cost-effectiveness of COVID-19 vaccine dose-fractionation strategies in India. We used large-scale survey data of the WTP together with data of vaccine and hospital admission costs to build the model, and we estimated the costs due to hospitalization and vaccination and the economic benefits due to reduction in COVID-19 deaths achieved with dose-fractionation strategies in India. When exploring a wide range of disease transmissibility and vaccine efficacy, we found that fractional doses of vaccines could be an economically viable vaccination strategy compared to the alternatives of either full-dose vaccination or no vaccination. We mainly analyzed the ChAdOx1 (Covishield) vaccine manufactured by the Serum Institute of India. The uncertain clinical relevance and effect of fractionation for many other vaccines raise an urgent need of having more research on the immunogenicity, safety and programmatic feasibility of fractionated doses of vaccines. Limitations of this study include the exclusion of reinfection of SARS-CoV-2 and the costs of the non-pharmacological interventions enacted to slow transmission. Incorporating these factors will allow for an update of our models to evaluate the ongoing booster vaccination in India.
Policy implication	Our analysis suggests that dose fractionation of vaccines is a cost-effective strategy for mitigating the COVID-19 pandemic and could reduce a large number of deaths in LMICs. Fractional doses of vaccines will accelerate population coverage and, thereby, augment responsive capacity in managing new SARS-CoV-2 variants with increased transmissibility or immune escape potential.

For each scenario, we estimated the health and economic outcomes of each fractional-dose strategy. For example, taking a 1/4 fractional-dose strategy with 72% vaccine efficacy against infection after the second dose, the median incremental cost is expected to be positive because the vaccine costs incurred outstrip averted hospitalization costs (Supplementary Table 3). Under a high-transmission scenario (*R*_*e*_ = 5), the optimal strategy of 1/8 fractionated dose would avert 4 million years of life lost (YLL) (95% credible interval (CrI): −9.44, 18.01) at a cost of USD $10.63 billion (95% CrI: 9.01, 12.05) (Supplementary Table 3). Under a low-transmission scenario (*R*_*e*_ = 1.2), the optimal strategy still suggests the 1/8 fractionated dose, which is expected to avert 11.34 million YLL (95% CrI: 0.35, 21.74) at a cost of USD $8.8 billion (95% CrI: 7.64, 9.97). We also assessed the robustness of our results with reduced vaccine efficacy against infection perhaps for new SARS-CoV-2 variants (Extended Data Figs. 3–5), which denotes slightly lower expected gain in the net monetary benefit (NMB).

Fractional dosing of vaccines in the context of global vaccine shortages has a great potential to mitigate the healthcare and economic burdens. Our cost-effectiveness analysis provides an indication of potential benefits for the population vaccinated with fractional doses in India. We provide a data-driven modeling approach to tailoring fractional-dosing strategies to local epidemiological conditions, using the ChAdOx1 vaccine as an example. Compared to the alternatives of either full-dose vaccination or no vaccination, fractional doses of ChAdOx1 vaccines would be an economically optimal vaccination strategy.

The advent of widespread booster dose programs in high- and middle-income countries to mitigate new SARS-CoV-2 variants, such as Omicron, will aggravate the constraints of vaccine supplies to low-income countries^18,20^. Global vaccine shortages and inequity of vaccine distributions are likely to remain a public health problem by mid-2022, compounded by challenges in vaccination distribution systems and uptake among target populations. To alleviate such constraints, vaccine dose fractionation could serve as a cost-effective strategy to reduce public health and socioeconomic burdens. Vaccines might not necessarily be administered with the same protocols as were tested in clinical trials, as exemplified by the ‘first dose first’ strategy adopted in the United Kingdom and Canada, where the administration of the first doses of COVID-19 vaccines were prioritized by delaying the second doses^19^. Therefore, dose fractionation could also be considered as a legitimate modification of the vaccine schedule, driven by an unmet public health need. Furthermore, the specific fractionation of doses can be optimized to balance the costs of vaccine rollout with the benefits of averting COVID-19 morbidity and mortality. However, the dose-sparing strategy might not help address the problem of shifting resources required by vaccination campaigns.

A dose-sparing strategy could help to boost vaccine supply and increase vaccination coverage and, thereby, gain public health and economic benefits locally and globally. The World Health Organization (WHO) has acknowledged the potential benefits of fractional-dose strategies^21^; however, further policy and operational research into the acceptability of such strategies in specific country settings, such as India, is needed. The WHO suggests that policy recommendations for the dose-sparing strategy should be made only after an extensive evidence review in terms of immunogenicity and safety^21^. The ethics and politics of using an untested fractionated vaccine dose in a lower-resource setting, when higher-resource settings have access to full doses of the vaccines, should also be considered. The use of fractional doses in lower-resource settings might have the potential to exacerbate global vaccine inequity if, in consequence, there is less global pressure to improve access to vaccines in the developing world. Given the uncertain clinical relevance and effect of fractionation for many vaccines, there are currently no low-income countries adopting dose-sparing strategies for their COVID-19 vaccines in use. However, fractionated doses of COVID-19 vaccines have already been used in some specific contexts. The US Food and Drug Administration has authorized a low-dose (1/3 full dose) COVID-19 vaccine made by Pfizer-BioNTech for children 5–11 years old^22,23^. The Moderna vaccine has an approved dosage for booster vaccination at 1/2 dose of the original vaccine for adults^24^. This suggests the urgent need of having more research on the immunogenicity and safety and on the programmatic feasibility of fractional doses of vaccines^25^, perhaps by prospective randomized trials. Using fractional doses could help to optimize the availability of vaccine doses, and the development of fractional dosing as a public health strategy that is well supported by scientific research will be a valuable pursuit applicable to diverse settings in current COVID-19 outbreaks as well as in future pandemics.

We expect that dose-fractionation strategies will be considered in India when clinical evidence and programmatic feasibility of vaccine fractional dosing become available. Given that the vaccine booster programs against the Omicron variant began in India in January 2022 (three million COVID-19 booster doses had been administered in India as of 14 January 2022 (ref. ^6^)), fractional dosing might be applied to the booster doses because, as of 14 January 2022, 64.8% of the entire Indian population had received at least one dose^6,26^. With more evidence on the effectiveness and durability of booster vaccination with full dose^27^, fractional doses of booster vaccines might be suitable for individuals with prior infection or for those who are fully vaccinated As such, fractional doses of booster vaccines might be suitable for individuals with prior infection or for those who are fully vaccinated.

**Extended Data Fig. 6:**
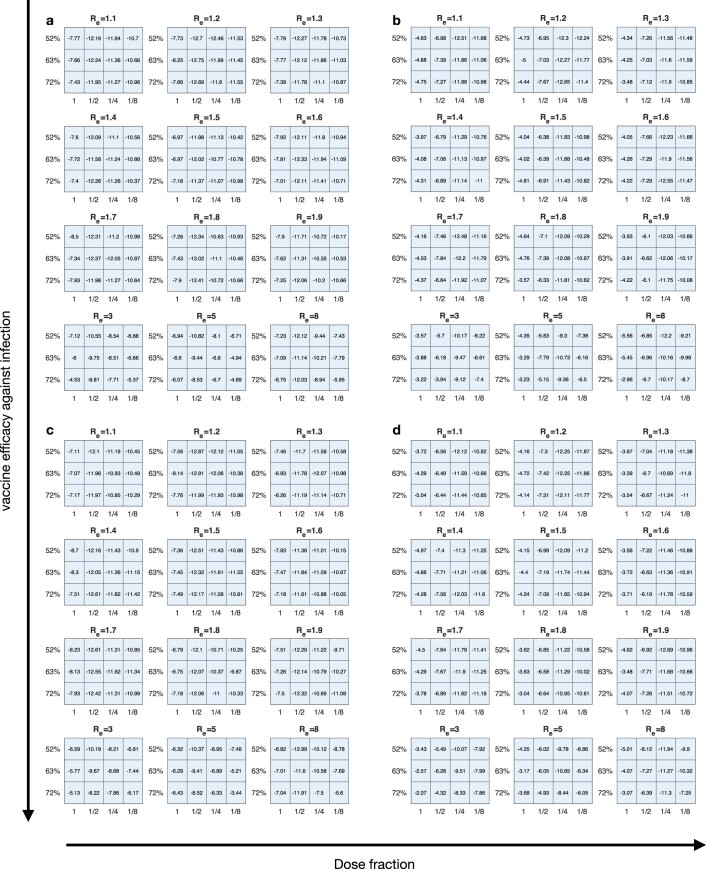
Sensitivity analysis on the expected gain in the NMB (2021 US dollars) for each fractional-dose strategy as compared to the status quo strategy (i.e., no vaccination) in India. Each panel from (a) to (d) corresponds to a test scenario from V1 to V4 (Supplementary Table 5), respectively. Vaccine cost is considered as in Supplementary Table 4, and the WTP per YLL averted are considered as US$1,097, US$1,251, US$2,977, US$3,150, and US$3,205 for the five age groups 0-5, 6-17, 18-49, 50-64, and > 65, respectively.

**Extended Data Fig. 7:**
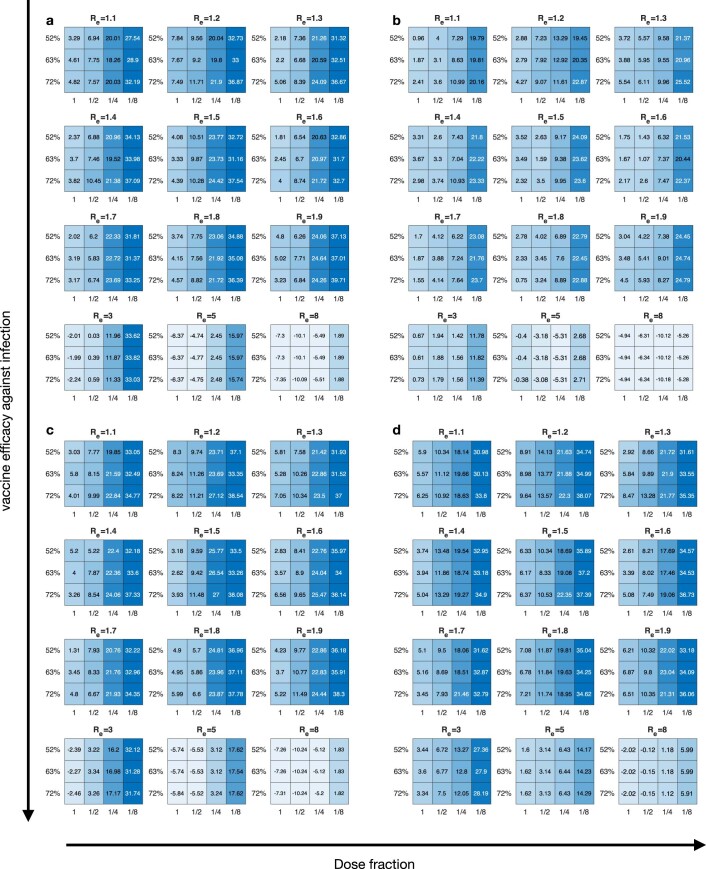
Sensitivity analysis on the expected gain in the NMB (2021 US dollars) for each fractional-dose strategy as compared to the status quo strategy (i.e., no vaccination) in India. Each panel from (a) to (d) corresponds to a test scenario from V5 to V8 (Supplementary Table 5), respectively. Vaccine cost is considered as in Supplementary Table 4, and the WTP per YLL averted are considered as US$1,097, US$1,251, US$2,977, US$3,150, and US$3,205 for the five age groups 0-5, 6-17, 18-49, 50-64, and > 65, respectively.

Concerns about vaccine resistance have also been raised as a potential disadvantage of dose-sparing strategies^25^. However, on the other hand, vaccines that protect against clinical disease appear to attenuate transmission, suggesting that increasing fractional dosing could reduce the attack rates^19^. Although we think that our qualitative results are robust and can be implemented, we underline several simplifying assumptions. First, our model does not explicitly include subgroups with anomalously high contact rates (for example, home caregivers), which could serve as viral reservoirs to disease spread. Second, our economic analysis considers only vaccination and hospitalization costs and the benefits of averting hospitalizations and deaths via vaccination. Future analysis would incorporate additional non-pharmaceutical interventions. Third, our model does not consider the reinfection of SARS-CoV-2, because the duration of immunity after natural infection or vaccination against SARS-CoV-2 might last for at least 6 months^28^. Fourth, in our economic calculations, we mainly considered the expense of vaccines in the private sector^29^ and hospitalization costs in private hospitals^30^. The vaccine cost can vary widely depending on both hospitals^29^ and vaccine types^31^, including the costs of transport and distribution^32^. The hospitalization cost is based on the report from an insurance company in India for the cost of COVID-19 treatment in private hospitals^30^, which might be much less in public hospitals^33^. Fifth, vaccine efficacy might have a wide range^31^. For example, the ChAdOx1 vaccine is estimated to have an efficacy of 62.1% (95% confidence interval: 41.0–75.7) against symptomatic COVID-19 for individuals receiving two standard doses^34^. There is still considerable uncertainty for the infection-blocking efficacy of vaccines, aside from the lack of data for the efficacy of low first dosing followed by standard second dosing. The variation in vaccine efficacy against infection might have a larger effect on the economic evaluation if patient time and salary loss during mandatory quarantine and hospitalization are included in the calculation of NMB. Sixth, our study considers the vaccine efficacy of a full dose against infection ranging from 52% to 72%, which might overestimate some vaccines used in India, especially for new variants. To account for these uncertainties, we provided comprehensive sensitivity analyses by changing basic assumptions, including the initial simulation conditions, vaccine efficacy against infection and the dosing strategy (Supplementary Table 4 and Extended Data Figs. 6 and 7). We found that, as a large proportion of individuals have been infected and recovered in India at the time of this writing, it might not be cost-effective to start the fractional-dose strategy at this moment unless the disease transmissibility becomes sufficiently high (such as for the Omicron variant; Extended Data Figs. 6 and 7). In addition, if a half dose followed by a full dose can provide similar post-second-dose immune responses as two full doses, the fractional-dose strategy would provide a higher expected gain in the NMB.

To summarize, fractionation of vaccine doses for SARS-CoV-2 is expected to be a cost-effective strategy for mitigating the lingering threat of the COVID-19 pandemic in India. If COVID-19 remains a persistent threat, especially with multiple SARS-CoV-2 variants escaping immunity, fractional dosing of vaccines might provide additional public health and economic benefits, especially when the global supply of vaccines is limited or in the early period of a new vaccine that is developed to target new variants in the future.

## Methods

### Epidemic model

We used a stochastic individual-based model to simulate the transmission of SARS-CoV-2 in a typical Indian community with 10,000 households for 150 days. To describe the individual’s infection and vaccination status, we assumed that each individual was in one of ten possible epidemiological states at any time (Extended Data Fig. 1). After exposure, an infected individual first experiences a non-infectious incubation period (on average of 1/*σ* days), after which this individual becomes pre-symptomatic with probability *p*_*sym*_ or becomes asymptomatic with probability 1−*p*_*sym*_. The asymptomatic cases are recovered at rate *γ*. The pre-symptomatic cases develop symptoms at rate ${\it{\epsilon }}$, after which they are recovered at rate *γ*. Supplementary Table 2 summarizes the parameterization of these parameters based on a series of references^6,11,36–50^.

The infectiousness of an infected individual depends on the infection status (that is, asymptomatic, pre-symptomatic or symptomatic) and the type of contacts (that is, household or non-household). The infectiousness of asymptomatic and pre-symptomatic cases is scaled by their relative infectiousness $\hat \omega$ and *ω*, respectively. We used the effective reproduction number (*R*_*e*_) to describe the disease transmissibility of SARS-CoV-2. In each simulation realization, we estimated *R*_*e*_ as the average number of secondary infections caused by the first 100 infectors. To calibrate the transmission rate *β* per contact for symptomatic cases, we used an interior point algorithm to minimize the mean square error between the targeted value of *R*_*e*_ and the mean estimation of *R*_*e*_ averaged over 100 realizations of the simulated pandemic To assess the effect of each vaccination strategy, each simulation realization first runs with no vaccination (that is, the status quo strategy) and then runs with the rollout of vaccines. Note that the status quo strategy assumes no vaccination across all 150 days. We explored two possible settings for the initialization of the simulated pandemic in India. The first one considered that only 2% of the Indian population had been randomly exposed on 16 January 2021 (that is, the start time of the Indian vaccination program^6^); the second one considered that 67.6% of the Indian population had been infected and recovered, with 22.0% and 5.3% of the Indian population having received the first standard dosing and the second standard dosing, respectively, on refs. ^6,21,51^ (Supplementary Table 4).

### SARS-CoV-2 vaccination program in India

We modeled the two-dose vaccination, with *d*_*dose*_ denoting the time interval between the first and second doses (Supplementary Table 2). Let *ω*_1_ and *ω*_2_ denote the vaccine efficacy against infection for the first and second doses, respectively (Supplementary Table 1). The immunity will be acquired with a delay of $d_{immunity}$ days after each dose. To reflect the vaccine efficacy against infection, the susceptibility of acquiring infection for vaccinated individuals is reduced by a factor *ω*_1_ after the first dose and by *ω*_1_ after the second dose. To reflect the vaccine efficacy against symptomatic disease, the probability of developing COVID-19 symptoms after infection for vaccinated individuals is reduced by a factor $\psi _1$ after the first dose and by $\psi _2$ after the second dose. To reflect the effect of vaccine breakthrough, we assume that, if vaccinated individuals acquire infection, they have the same transmission rate as those who are unvaccinated.

We considered two possible settings for the initialization of the Indian vaccination program. The first one assumed that the daily vaccination rate of the first dose was 0.01% of the Indian population on 16 January 2021 (ref. ^6^) (that is, the start time of the Indian vaccination program); the second one assumed that 22.0% and 5.3% of the Indian population had received the first standard dosing and the second standard dosing, respectively, by 11 July 2021 (ref. ^6^) (that is, based on the fourth survey of the Indian Council of Medical Research^51^). Through the 150-day simulation, in total 12.5% of the Indian population will be newly vaccinated, if the standard dosing is used for vaccinating each dose. We assigned the vaccines available per day to individuals requiring their first or second dose (that is, daily available vaccination number $\nu$; Supplementary Table 2). Because people over age 65 were prioritized for vaccination, we assumed that vaccines were first delivered randomly to people over age 65, after which adults 17–64 years of age started to receive their first dosing. Individuals receiving the first dosing will receive the second dosing after $d_{dose}$ days.

### Individual-based network

We considered each Indian community consisting of 10,000 households with 47,568 individuals. In each Indian community, individuals are connected via a static contact network. We considered five age groups: 0–5, 6–17, 18–49, 50–64 and >65 years. The size of each household was sampled using the empirical distribution with mean as 4.76 and standard deviation as 1.74, according to the 2011 Census Data in India^52^. Members within each household are fully connected (that is, each individual can contact other members in the same household). Following Du et. al.^45^, we randomly connected individuals from different households according to the data of age-specific contact rates in India^53^. To determine the number of contacts between an individual of age group *a*_*i*_ and another individual of age group *a*_*j*_, we drew a random number using a Poisson distribution with rate as the mean number of contacts between age groups *a*_*i*_ and *a*_*j*_. In our model, each Indian community captured the characteristics of the household structure and contact patterns in India, so we obtained the economic evaluation results for the whole of India by scaling those obtained from a single community with 47,568 individuals to the 1,366 million Indian population.

### Study perspective

Our economic calculations have a restricted scope. The cost of implementing each transmission and vaccination scenario is derived from an Indian health sector perspective. This includes costs to both government and patients (including vaccine and hospitalization costs) but excludes the patient salary loss during hospitalization and indirect costs (including costs to other household members and the employers). To quantify public health benefits, we considered the economic benefits of averting mortality and the cost savings of averting hospital admissions, but we did not consider the prevention of non-fatal morbidity caused by SARS-CoV-2 infection (which might be substantial) and the indirect health and mental health consequences of the pandemic. Finally, we assessed vaccination strategies across a wide range of reproduction numbers, but we did not account for the direct or indirect costs of the non-pharmacological interventions enacted to slow transmission in the milder scenarios.

### Estimating the YLL averted and monetary costs

Given each transmission and vaccination scenario, we simulated 100 random realizations for each candidate vaccination strategy (including the status quo), with costs including the vaccination cost and hospital admission cost due to the COVID-19 and YLLs averted considering mortality due to COVID-19. For each realization, we determined the YLL averted for each strategy $\tau$ as follows:

1. Calculate the difference in deaths for age group *a* as ${\Delta}_{a,\tau } = D_{a,0} - D_{a,\tau }$, where $D_{a,0}$ and $D_{a,\tau }$ are the numbers of total death in age group *a* simulated by the status quo and strategy $\tau$, respectively.
2. Estimate the YLL averted by the vaccination strategy $\tau$ as

$$B_\tau = {\Sigma}_aB_{\tau ,a} = {\Sigma}_a(\lambda _a - a){\Delta}_{a,\tau }$$

where *λ*_*a*_ denotes the future-discounted life expectancy for individuals of age *a*.

Similarly, we determined the incremental monetary costs for each strategy *τ* as compared to the status quo by:

$$C_\tau = (T_\tau - T_0)c_T + {\Sigma}_ac_{H,a}(H_{\tau ,a} - H_{0,a})$$

where $T_\tau - T_0$ is the difference in the total number of administered vaccines between the strategy *τ* and status quo; *c*_*T*_ is the cost per dose delivered for vaccine procurement price plus vaccination delivery costs combined; $H_{\tau ,a} - H_{0,a}$ is the difference in the total number of hospitalizations for age group *a* between the strategy *τ* and status quo; and $c_{H,a}$ is the median COVID-19 hospitalization cost for age group *a*. Detailed values of these cost parameters are given in Supplementary Table 5.

### Estimating the WTP per averted YLL and the cost-effectiveness

The WTP per averted YLL is the maximum price a society is willing to pay to prevent the loss of 1 year of life. We used large-scale survey data from the Centre for Monitoring Indian Economy’s Consumer Pyramids Household Survey^35^, which is population-representative and has surveyed more than 174,000 households (>0.85 million individuals) in India. To perform the estimation, we first obtained the 2019 household-level mean monthly consumption from these data. We then assigned the household consumption to each age group using the Organisation for Economic Co-operation and Development (OECD)’s modified formula, with each child in the household assigned with a weight of 0.3 and each adult in the household assigned with a weight of $$\frac{{0.5 \ast (n + 1)}}{n}$$, where *n* is the number of adults in the household. Each household member is assigned with household consumption according to the corresponding weight. By aggregating the household consumption from the individual level into the national level according to OECD’s sampling weights, we estimate that the WTP per averted YLL in India is USD $1,097, USD $1,251, USD $2,977, USD $3,150 and USD $3,205 for the five age groups, 0–5, 6–17, 18–49, 50–64 and >65 years, respectively.

Given the WTP per averted YLL (*θ*_*a*_ for age group *a*), we calculated the NMB of a strategy as

$$NMB_\tau = {\Sigma}_a\theta _a \ast B_{\tau ,a} - C_\tau$$

The costs and YLLs averted are all scaled using the Indian population of 1,366 million individuals. Our estimation examines a wide range of possible disease transmissibility and vaccine efficacy. Given each set of disease transmissibility and vaccine efficacy, (1) we first simulated 100 stochastic realizations for each candidate vaccination strategy (including the status quo); then (2), with each realization, we used the above formulas to calculate the expected gain in the NMB for each candidate strategy *τ* as compared to the status quo strategy; after which (3), we identified the winning strategy as the strategy *τ* giving the highest gain in the NMB. We then calculated the proportion that each strategy *τ* is selected as the winning strategy across all 100 simulation realizations. Finally, we chose the optimal strategy as the one having the highest chance to serve as the winning strategy.

### Sensitivity analysis on the WTP per averted YLL

We conducted several sensitivity analyses on the WTP per averted YLL. (1) The 2020 gross domestic product per capita purchasing power parity (PPP) is USD $6,454.3 and USD $63,543.6 in India and the United States, respectively^48^. Health economists have inferred from healthcare expenditure that the United States is willing to pay USD $100,000 per quality-adjusted life-year (QALY)^49^, in which YLL is a key component. The PPP is a popular metric of economists that uses the prices of specific goods to compare the economic productivity and standards of living among different countries, which is used to estimate the WTP in many applications (for example, the New Rural Cooperative Medical Scheme in China^50^). We, thus, assume that the WTP in India is USD $10,517 per averted YLL, which is estimated by scaling the WTP per averted YLL in the United States according to the difference in PPP between India and the United States. (2) We performed two additional sensitivity analyses: (i) using the WTP per averted YLL estimated for middle-income country settings (that is, USD $5,089 per QALY in Thailand^51^) and (ii) using the WTP per averted YLL estimated for high-income country settings (that is, USD $100,000 per QALY in the United States^49^).

### Statistical analyses

We estimated the expected gain in the NMB for each tested vaccination strategy in India (that is, using full dosing, 1/2 dosing, 1/4 dosing or 1/8 dosing) by using 100 stochastic simulations, with the resulting NMBs used to estimate the median and 95% CrI. All analyses were completed using MATLAB 2020a.

### Reporting Summary

Further information on research design is available in the Nature Research Reporting Summary linked to this article.

## Online content

Any methods, additional references, Nature Research reporting summaries, source data, extended data, supplementary information, acknowledgements, peer review information; details of author contributions and competing interests; and statements of data and code availability are available at 10.1038/s41591-022-01736-z

## Supplementary information

Supplementary Information Supplementary Tables 1–5

Reporting Summary

## Data Availability

All data were collected from open-access sources with detailed description in the Methods. All data used in this study are publicly available, including the daily vaccination rate of the first dose in India (https://ourworldindata.org/coronavirus-testing), the 2011 Census Data in India (https://censusindia.gov.in/Tables_Published/HH-Series/hh_series_tables_20011.html) and the data from the Centre for Monitoring Indian Economy’s Consumer Pyramids Household Survey (https://consumerpyramidsdx.cmie.com/).

## Code availability

All codes used in this study can be accessed at https://github.com/ZhanweiDU/VaccineFractionation
