# Supplementary material for: Modeling comparative cost-effectiveness of SARS-CoV-2 vaccine dose fractionation in India
Source: Nat Med. 2022 Feb 24;28(5):934–8. doi: 10.1038/s41591-022-01736-z (PMC9117137; doi:10.1038/s41591-022-01736-z)
Supplement: Supplementary file 1 — Supplementary Tables 1–5 [file 41591_2022_1736_MOESM1_ESM.pdf]

nature portfolio

https://doi.org/10.1038/s41591-022-01736-z

Supplementary information

Modeling comparative cost-effectiveness of SARS-CoV-2 vaccine dose fractionation in India

In the format provided by the authors and unedited

Supplementary Tables

Supplementary Table 1. Vaccine efficacy against infection and vaccine efficacy against symptomatic disease for the ChAdOx1 (Covishield, Astrazeneca) vaccine manufactured by the Serum Institute of India.

Parameters	Values	Data source
ω_1: vaccine efficacy against infection , i.e., infection-blocking vaccine efficacy (%) during the period between the first dose and the second dose	46.2%	Mean estimate for the vaccine effectiveness of a single dose of ChAdOx1 vaccine against SARS-CoV-2 infection[55]
ω_2: vaccine efficacy against infection , i.e., infection-blocking vaccine efficacy (%) after the second dose	Assumed to be 51.5%, 63.1%, and 72.1% for different settings of dosing, respectively	Estimated vaccine effectiveness against SARS-CoV-2 infection for individuals fully vaccinated with two doses of ChAdOx1 vaccine: 63.1% (95% CI: 51.5, 72.1) [55]
ψ_1: vaccine efficacy against symptomatic disease , i.e., symptom-blocking vaccine efficacy (%) during the period between the first dose and the second dose	79.2%	Mean estimate for the effectiveness of a single dose of ChAdOx1 vaccine against moderate-to-severe disease[55]
ψ_2: vaccine efficacy against symptomatic disease , i.e., symptom-blocking vaccine efficacy (%) after the second dose	Assumed to be 79.2%, 81.5%, and 99% for different settings of dosing	Estimated vaccine effectiveness against moderate-to-severe disease for individuals fully vaccinated with two doses of ChAdOx1 vaccine: 81.5% (95% CI: 9.9, 99)[55]

Supplementary Table 2. Epidemiological parameters for the individual-based SARS-CoV-2 infection dynamic model. We model multiple strategies for accelerating vaccination rollout combined with a fixed baseline level with no vaccination (i.e., *status quo*).

**Parameters**	**Values**	**Data source**
N: the total number of individuals in 10,000 households	47,568	Assumed
β: transmission rate per contact for a symptomatic case	Calibrated to R_e	
Initial number of the exposed individuals	10	Assumed
σ: transition rate out of exposed state	1/3	[47]
γ: recovery rate of symptomatic individuals	1/4	[43,45]
$\hat{\gamma}$: recovery rate of asymptomatic individuals	1/9	[56]
ϵ: transition rate from the pre-symptomatic to the symptomatic stage	1/2	[47]
ω: relative infectiousness of pre-symptomatic cases as compared to symptomatic cases	1.57	[43,45]
$\hat{\omega}$: relative infectiousness of asymptomatic cases as compared to symptomatic cases	0.5	[44]
p_{sym}: proportion of infections that are symptomatic	75%	[42,43]
γ^H: recovery rate in hospitalized compartment	0.143	[41]
h_a: age-specific proportion of symptomatic cases that are hospitalized	[0%, 0.025%, 2.672%, 9.334%, 15.465%] for [0-4y, 5-17y, 18-49y, 50-64y, >65y]	[39]
η: transition rate from symptomatic to hospitalized	1/5.9	[40]
μ_a: age-specific mortality rate for hospitalized cases	[3.9%, 12.1%, 3.0%, 10.5%, 22.7%] for [0-4y, 5-17y, 18-49y, 50-64y, >65y]	[38,39]
γ_d: transition rate from hospitalized to deceased for cases that succumb	0.128	[38]
γ_h: transition rate from hospitalized to recovered for cases discharged alive	0.091	[38]
λ_a: life expectancy (years) for age group a, adjusted assuming a 3% yearly discount rate	[29.4, 28.1, 23.2, 16.3, 10.2] for [0-4y, 5-17y, 18-49y, 50-64y, >65y]	[37]

***Vaccination* parameters**		
v: daily available vaccination number	The reported nationwide daily vaccination rate of the first standard dose in India	Assuming that the Indian population is 1,366 million in 2021 and the nationwide daily vaccination rate of the first standard dose was 0.01% of the Indian population on January 16, 2021 and was increasing to 0.25% by the end of the study period [36], the nationwide cumulative vaccine coverage of the first dosing accounts for 0.99%, 7.17%, and 12.5% of the Indian population through the first 50, 100, and 150 days of the study period [36]. The number of vaccinations on each day is calculated as the product of the daily vaccination rate and the Indian population size. For the *1/f* fractionated doses, the daily vaccination rate is f - 1 times higher than that of the standard dose.
w_v: number of weeks for vaccination assignment	20	[48]
d_{dose}: days between the first and second doses	28	[11]
d_{immunity}: days acquiring immunity after the first or second dose	14	[49,50]
φ: vaccine adherence rate, which is the maximum uptake rate by each age group. The first and second dosing are assumed to have the same vaccine adherence rate.	0.7	[51]
***Status quo* parameters**		
v: daily available vaccination number assuming an India population of 1366 million and a daily vaccination rate of 0%	0	Assumed

Supplementary Table 3. The median (95% CrI) estimates of the incremental costs, YLL averted, and the expected gain in the net monetary benefits (NMB) for the 1/8 fractional-dose strategy (i.e., the optimal strategy). The baseline strategy to compare with is the status quo strategy (i.e., no vaccination) in India. The vaccine efficacy against infection is considered as 72%, and the vaccine efficacy against symptomatic disease is considered as 99% [55]. The estimation examines a wide range of possible transmission scenarios, with *Re* increasing from 1.1 to 8.All estimates are scaled to represent the estimation for the whole India population of 1,366 million.

R_e	Median (95% CrI) of the incremental costs (billion 2021 US dollars)	Median (95% CrI) of the incremental YLL averted (in million)	Median (95% CrI) of the expected gain in the NMB (billion 2021 US dollars)
1.1	8.79 (95% CrI:7.5, 9.88)	9.67 (95% CrI:-1.32, 19.69)	21.94 (95% CrI:-10.6, 52.3)
1.2	8.8 (95% CrI:7.64, 9.97)	11.34 (95% CrI:0.35, 21.74)	27.05 (95% CrI:-7.74, 63.85)
1.3	8.99 (95% CrI:7.62, 10.42)	10.93 (95% CrI:0.13, 23.08)	26.36 (95% CrI:-8.25, 62.34)
1.4	9 (95% CrI:7.49, 10.3)	10.33 (95% CrI:-1.64, 22.19)	23.21 (95% CrI:-14.86, 61.38)
1.5	8.99 (95% CrI:7.85, 10.19)	11.51 (95% CrI:-1.27, 22.35)	26.46 (95% CrI:-11.25, 60.84)
1.6	9.02 (95% CrI:7.49, 10.61)	11.05 (95% CrI:-1.05, 21.93)	27.18 (95% CrI:-15.91, 59.78)
1.7	9.19 (95% CrI:7.64, 10.78)	9.54 (95% CrI:-3.5, 21.1)	22.27 (95% CrI:-19.49, 57.81)
1.8	9.18 (95% CrI:7.64, 10.26)	10.11 (95% CrI:-4.1, 22.86)	25.45 (95% CrI:-20.14, 63.77)
1.9	9.3 (95% CrI:7.85, 10.54)	10.19 (95% CrI:-3.74, 23.7)	22.91 (95% CrI:-20.42, 65.78)
3	9.82 (95% CrI:8.49, 11.27)	8.87 (95% CrI:-3.91, 21.77)	19.07 (95% CrI:-22.3, 58.79)
5	10.63 (95% CrI:9.01, 12.05)	4 (95% CrI:-9.44, 18.01)	1.78 (95% CrI:-41.42, 43.88)
8	10.84 (95% CrI:9.25, 12.36)	1.03 (95% CrI:-11.88, 14.2)	-7.76 (95% CrI:-49.49, 34.11)

Supplementary Table 4. Sensitivity analyses for the SARS-CoV-2 vaccination settings and initial conditions of simulations. Each scenario from V1 to V8 changes one, two, or three basic assumptions given in the appendix (**Supplementary Table S3**), as indicated in the second column.

Scenarios	Settings
V1	Alternative initial conditions*<br>Vaccines without the infection-blocking efficacy[+]<br>The first dosing is half of the second dosing[#]
V2	Alternative initial conditions<br>Vaccines without the infection-blocking efficacy
V3	Alternative initial conditions<br>The first dosing is half of the second dosing
V4	Alternative initial conditions
V5	Vaccines without the infection-blocking efficacy<br>The first dosing is half of the second dosing
V6	Vaccines without the infection-blocking efficacy
V7	The first dosing is half of the second dosing
V8	Cost of administering each *1/f* fractionated dose of vaccination (c_T) [^]: \$12/*f*

* Alternative initial conditions in simulations: The results of the fourth survey of Indian Council of Medical Research (ICMR), which was conducted during June 21 to July 11, 2021, show that the seropositivity was 67.6% in India during that time [52]. In addition, 22.0% and 5.3% of the Indian population had received the first dosing and second dosing on July 11, 2021, respectively [36]. We thus assume an alternative initial condition with 67.6% of the Indian population having been infected and recovered, with 22.0% and 5.3% of the Indian population having received the first standard dosing and second standard dosing, respectively.

[+] We assume that the vaccine efficacy against infection (ω_1 and ω_2) after each dose does not impact the susceptibility of acquiring infection, i.e., $\omega_1 = \omega_2 = 0$

[#] In the Phase 3 trial of the ChAdOx-1 S (recombinant) vaccine, an initial half-dose showed a lower immune response than a full dose, whereas a half-dose followed by a full-dose gave similar post-second dose immune responses as two full doses [21]. Accordingly, we assume that individuals receiving half of the second dose for the first dose will have the same vaccine efficacies against infection and symptomatic disease. For example, given the 1/4 fractionalized dosing, this will be the 1/8 dosing for the first dose followed by 1/4 dosing for the second dose.

[^] The cost of administering each *1/f* fractionated dose of vaccine (c_T) is considered as \$12/*f*, which indicates that the delivery cost can also be divided.

Supplementary Table 5. Cost parameters of the ChAdOx1 (Covishield, Astrazeneca) vaccine manufactured by the Serum Institute of India

Parameter	Value (2021 US dollars)
c_T: cost of administering each standard dose	$12 [29] as cost per dose delivered for vaccine procurement price ($3) plus vaccination delivery costs ($9) combined
c_T: cost of administering each *1/f* fractionated dose	$(3/*f* + 9), where $3 is the vaccine procurement price per dose and $9 accounts for the vaccination delivery costs including delivery, distribution, and potential wastage [32]. We assume the cost of administering each *1/f* fractionated dose of vaccination (c_T) to be $(3/*f* + 9).
$c_{H,a}$: median COVID-19 hospitalization cost by age group	$604 [30] for any age group
