# Supplementary material for: Modeling comparative cost-effectiveness of SARS-CoV-2 vaccine dose fractionation in India
Source: Nat Med. 2022 Feb 24;28(5):934–8. doi: 10.1038/s41591-022-01736-z (PMC9117137; doi:10.1038/s41591-022-01736-z)
Supplement: Supplementary file 2 — Reporting Summary [file 41591_2022_1736_MOESM2_ESM.pdf]

nature research

Corresponding author(s): Benjamin J. Cowling

Last updated by author(s): Jan 14, 2022

Reporting Summary

Nature Research wishes to improve the reproducibility of the work that we publish. This form provides structure for consistency and transparency in reporting. For further information on Nature Research policies, see our Editorial Policies and the Editorial Policy Checklist.

Statistics

For all statistical analyses, confirm that the following items are present in the figure legend, table legend, main text, or Methods section.

n/a	Confirmed	
☐	☒	The exact sample size (n) for each experimental group/condition, given as a discrete number and unit of measurement
☐	☒	A statement on whether measurements were taken from distinct samples or whether the same sample was measured repeatedly
☒	☐	The statistical test(s) used AND whether they are one- or two-sided<br>*Only common tests should be described solely by name; describe more complex techniques in the Methods section.*
☐	☒	A description of all covariates tested
☐	☒	A description of any assumptions or corrections, such as tests of normality and adjustment for multiple comparisons
☐	☒	A full description of the statistical parameters including central tendency (e.g. means) or other basic estimates (e.g. regression coefficient) AND variation (e.g. standard deviation) or associated estimates of uncertainty (e.g. confidence intervals)
☒	☐	For null hypothesis testing, the test statistic (e.g. F, t, r) with confidence intervals, effect sizes, degrees of freedom and P value noted<br>*Give P values as exact values whenever suitable.*
☒	☐	For Bayesian analysis, information on the choice of priors and Markov chain Monte Carlo settings
☐	☒	For hierarchical and complex designs, identification of the appropriate level for tests and full reporting of outcomes
☒	☐	Estimates of effect sizes (e.g. Cohen's d, Pearson's r), indicating how they were calculated

Our web collection on statistics for biologists contains articles on many of the points above.

Software and code

Policy information about availability of computer code

Data collection	No software used
Data analysis	All codes for simulation, parameter estimation and statistical analyses were completed using Matlab R2020a. Related codes and data are available in Github for reproduction (https://github.com/ZhanweiDU/VaccineFractionation).

For manuscripts utilizing custom algorithms or software that are central to the research but not yet described in published literature, software must be made available to editors and reviewers. We strongly encourage code deposition in a community repository (e.g. GitHub). See the Nature Research guidelines for submitting code & software for further information.

Data

Policy information about availability of data

All manuscripts must include a data availability statement. This statement should provide the following information, where applicable:

- Accession codes, unique identifiers, or web links for publicly available datasets
- A list of figures that have associated raw data
- A description of any restrictions on data availability

All data are collected from open source with detailed description in Section Method. All data used in the manuscript are publicly available, including the daily vaccination rate of the first dose in India (https://ourworldindata.org/coronavirus-testing), the 2011 Census Data in India (https://censusindia.gov.in/Tables_Published/HH-Series/hh_series_tables_20011.html), and the data from the Centre for Monitoring Indian Economy's Consumer Pyramids Household Survey (https://consumerpyramidsdx.cmie.com/).

Field-specific reporting

Please select the one below that is the best fit for your research. If you are not sure, read the appropriate sections before making your selection.

☒ Life sciences ☐ Behavioural & social sciences ☐ Ecological, evolutionary & environmental sciences

For a reference copy of the document with all sections, see nature.com/documents/nr-reporting-summary-flat.pdf

Life sciences study design

All studies must disclose on these points even when the disclosure is negative.

Sample size	no sample-size calculation was performed. We generate 100 values of net monetary benefit (NMB) by simulations for each strategy in our study, which is large enough to estimate the median and 95% credible interval of NMB.
Data exclusions	no data were excluded
Replication	All simulations at replication were successful. We fix the seed for the MATLAB® random number generator in each simulation and can perform the same results in repeated simulations.
Randomization	this is not relevant to our study, giving we simulate by computer for every agents
Blinding	this is not relevant to our study, giving we simulate by computer for every agents

Reporting for specific materials, systems and methods

We require information from authors about some types of materials, experimental systems and methods used in many studies. Here, indicate whether each material, system or method listed is relevant to your study. If you are not sure if a list item applies to your research, read the appropriate section before selecting a response.

Materials & experimental systems

n/a	Involved in the study
☒	☐ Antibodies
☒	☐ Eukaryotic cell lines
☒	☐ Palaeontology and archaeology
☒	☐ Animals and other organisms
☒	☐ Human research participants
☒	☐ Clinical data
☒	☐ Dual use research of concern

Methods

n/a	Involved in the study
☒	☐ ChIP-seq
☒	☐ Flow cytometry
☒	☐ MRI-based neuroimaging
